# Platelets from patients with myeloproliferative neoplasms have increased numbers of mitochondria that are hypersensitive to depolarization by thrombin

**DOI:** 10.1038/s41598-023-36266-2

**Published:** 2023-06-06

**Authors:** David M. Ross, Hai Po Helena Liang, Zeenet Iqra, Shane Whittaker, Chuen Wen Tan, Brian J. Dale, Vivien M. Chen

**Affiliations:** 1 Centre for Cancer Biology, SA Pathology and University of South Australia, Adelaide, Australia; 2 Department of Haematology, Royal Adelaide Hospital, 6E359, Port Rd, Adelaide, SA 5000 Australia; 3 Department of Haematology, Flinders University and Medical Centre, Adelaide, Australia; 4 Adelaide Medical School, University of Adelaide, Adelaide, Australia; 5 ANZAC Research Institute, Concord Repatriation General Hospital, Sydney, NSW Australia; 6 Clinical and Health Sciences, University of South Australia, Adelaide, Australia; 7 Department of Haematology, Concord Repatriation General Hospital, Sydney, NSW Australia; 8 Faculty of Medicine and Health, Concord Clinical School, University of Sydney, Sydney, NSW Australia

**Keywords:** Haematological cancer, Mitochondria, Thromboembolism

## Abstract

Thrombosis is one of the cardinal manifestations of myeloproliferative neoplasms (MPN). The mechanisms leading to a prothrombotic state in MPN are complex and remain poorly understood. Platelet mitochondria play a role in platelet activation, but their number and function have not been extensively explored in MPN to date. We observed an increased number of mitochondria in platelets from MPN patients compared with healthy donors. MPN patients had an increased proportion of dysfunctional platelet mitochondria. The fraction of platelets with depolarized mitochondria at rest was increased in essential thrombocythemia (ET) patients and the mitochondria were hypersensitive to depolarization following thrombin agonist stimulation. Live microscopy showed a stochastic process in which a higher proportion of individual ET platelets underwent mitochondrial depolarization and after a shorter agonist exposure compared to healthy donors. Depolarization was immediately followed by ballooning of the platelet membrane, which is a feature of procoagulant platelets. We also noted that the mitochondria of MPN patients were on average located nearer the platelet surface and we observed extrusion of mitochondria from the platelet surface as microparticles. These data implicate platelet mitochondria in a number of prothrombotic phenomena. Further studies are warranted to assess whether these findings correlate with clinical thrombotic events.

## Introduction

The classic Philadelphia-negative myeloproliferative neoplasms (MPNs)—essential thrombocythemia (ET), polycythemia vera (PV), and primary myelofibrosis (PMF)—are clonal hematopoietic disorders characterized by excess circulating platelets, leukocytes or erythrocytes in which much of the morbidity is due to inflammation, thrombosis, and hemorrhage. The *JAK2* V617F mutation that activates JAK-STAT signalling is present in around 95% of patients with PV and 50–60% of patients with ET or PMF. Alternative JAK-STAT activating mutations in *JAK2*, *MPL*, or *CALR* are present in the majority of the remaining cases.

The normal function of platelets is primary hemostasis, that is, to stop blood loss after vascular damage. However, platelet function is altered in prothrombotic pathological conditions^1–3^. Thrombocytosis is common in MPN and is a diagnostic criterion for ET. Despite this, quantitative excess of platelets has not been found to correlate with the increased thrombosis risk. In the UK Medical Research Council PT-1 study the majority of vascular events occurred in people with normal or mildly elevated platelet counts^4^. In an Italian retrospective study, the occurrence of thrombosis prior to MPN diagnosis was more frequent in individuals with platelet counts ≤ 700 × 10^9^/L than in those with higher counts^5^. Patients with ET and a *CALR* mutation have higher average platelet counts, but a lower risk of thrombosis^6, 7^. These observations suggest that qualitative changes in platelet function may be more important determinants of thrombosis risk than a quantitative excess. Consistent with this, MPN platelets support increased thrombin generation showing a dose-dependent increase with *JAK2* V617F allele burden^8^, and are hypersensitive to low dose agonists^3^.

Mitochondria play an important role in platelet hemostatic function. Platelet activation leads to mitochondrial membrane depolarization, calcium flux into the cytoplasm. Opening of the mitochondrial permeability transition pore and loss of inner mitochondrial membrane potential precede surface membrane expression of phosphatidylserine, and procoagulant platelet formation^9, 10^. In addition, it has been demonstrated that cell-free mitochondria, shed from cells as microparticles, can elicit a potent inflammatory response that may contribute to thrombotic risk following brain injury or blood transfusion^11, 12^. Whether perturbation of mitochondrial platelet function is clinically important in MPN-related thrombosis is not yet established.

We compared mitochondria in platelets from MPN patients with healthy donors to test whether altered platelet mitochondrial number or function might play a role in the hemostatic alterations observed in MPN.

## Results

We collected samples from 89 MPN patients and 41 healthy donors. Samples for each set of experiments were taken from suitable sequentially consented patients from outpatient clinics and contemporaneously recruited controls. The median age of the healthy donors was 46 years (range, 20–68) and 28 were female (68%). The MPN patient characteristics are summarized in Supplementary Table S1 online. The median age was 65 years (range, 30–96) and 46 patients were female (52%). The most frequent MPN diagnosis was ET (57/89 patients). The median platelet count was 452 × 10^9^/L, reflecting the use of cytoreductive treatment in 64% of patients, predominantly hydroxyurea. Anti-thrombotic therapy was used in 93% of patients, predominantly aspirin monotherapy (70%). Twenty-seven patients had a history of prior venous or arterial thrombosis (two patients had missing data). Sixty patients had the *JAK2* V617F mutation and 15 patients had a *CALR* mutation. The median variant allelic fraction (VAF) of *JAK2* V617F in those patients with a quantitative result was 28% (0.7–100%; n = 53). In 29 *JAK2* ET patients the median VAF was 18% (range, 0.7–47%). Among the ET patients, 41 were considered high risk (age > 60 years and/or prior thrombotic event) and 12 were low risk (4 patients had missing data).

**Figure 1:**
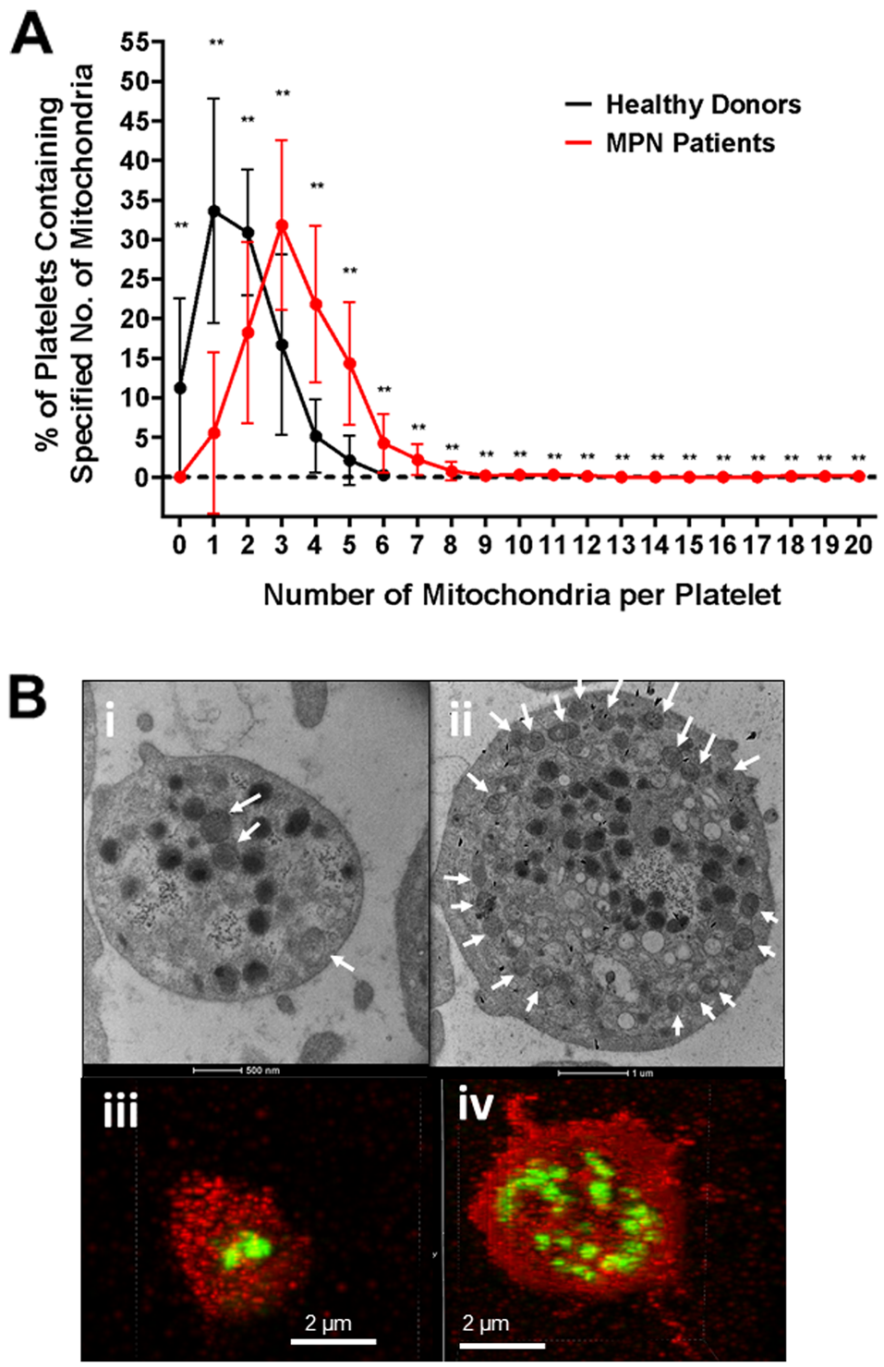
Increased mitochondria in platelets of MPN patients. (**A**) TEM images were used to count the number of mitochondria within 50 platelets per individual subject. A comparison between healthy donors (n = 16) and MPN patients (n = 23) of the number of mitochondria per platelet was done using categorized data ranging from 0 mitochondria/platelet to 20 mitochondria/platelet. Error bars at each point represent differences between individual subjects. (**B**) TEM images of 3 mitochondria (indicated with white arrows) in a healthy donor platelet (i) and 20 mitochondria in an ET patient platelet (ii). Confocal microscopy images of 2 mitochondria (in green) in a healthy donor platelet (iii) and 20 mitochondria in an ET patient platelet (iv).

The number of mitochondria within 50 platelets from each individual subject was counted by visual inspection of TEM images. The median number of mitochondria per platelet was 3 (range, 1–20) in 23 MPN patients versus 2 (range, 0–6) in 16 normal donors. The number of mitochondria per platelet was more variable in MPN patients than in normal donors (Fig. 1A), with some MPN patients having a subpopulation of platelets that showed substantially increased numbers of mitochondria. Based on the upper range in the healthy donors, we considered platelets with > 6 mitochondria to have increased mitochondrial number. Platelets with increased mitochondria were seen in 0/16 healthy donors versus 19/23 (83%) of MPN patients. Counting mitochondria by confocal microscopy within the same cohort yielded similar results (Supplementary Fig. S1 online). Representative TEM and confocal microscopy images (Fig. 1B) illustrate differences between healthy donors and MPN patients.

The median number of mitochondria counted in 50 MPN platelets in TEM images did not differ according to mutation status: *JAK2* V617F mutation (n = 14 patients) 180 mitochondria (range 99–204); versus *CALR* mutation (n = 4 patients) 188 mitochondria (range 183–220); versus triple-negative (n = 5 patients) 201 mitochondria (range 109–208). The *JAK2* V617F VAF (measured in leukocytes) was not correlated with the number of platelet mitochondria (r = 0.31, *P* = 0.35). Mitochondrial number was not correlated with age, neither in healthy donors nor in MPN patients (Supplementary Fig. S2 online). There was no sex difference in mitochondrial number in healthy donors. Female MPN patients had higher mitochondrial number than males, but this difference was possibly due to an imbalance in MPN subtypes, with higher numbers in ET patients who were predominantly female (Supplementary Fig. S2 online).

**Figure 2:**
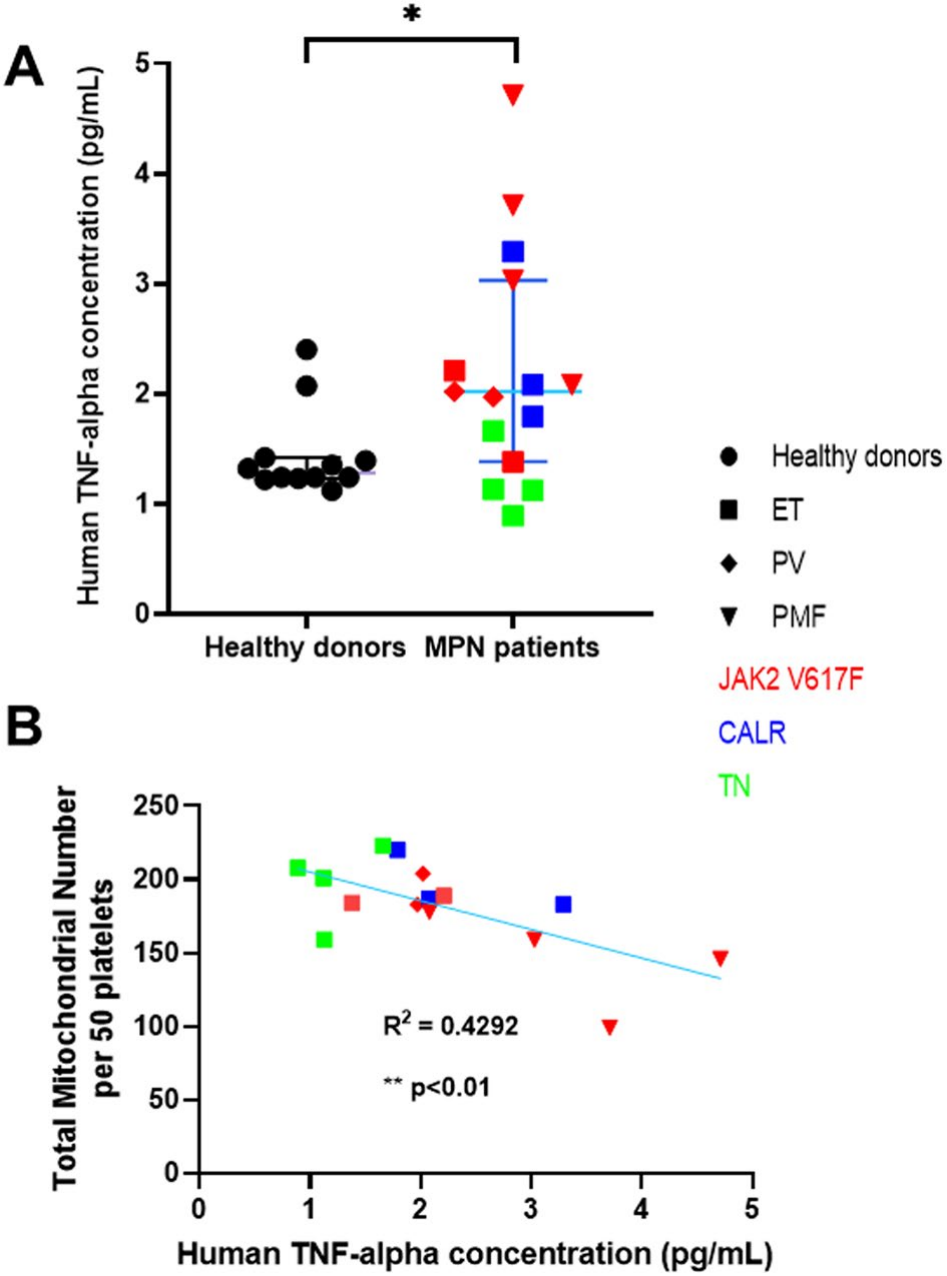
TNFα is increased in MPN patients and inversely correlated with platelet mitochondrial mass. (**A**) The concentration of TNFα measured in plasma samples was higher in MPN patients than in healthy donors (median and 95% CI). (**B**) In MPN patients there was an inverse correlation between TNFα concentration and platelet mitochondrial mass (assessed as total number of mitochondria in 50 platelets in TEM images). Healthy donors (black circles), ET (squares), PV (diamonds), PMF (triangles), JAK2 V617F (red), CALR (blue), triple negative (green). *P* < 0.05 (*), Pearson correlation, *P* < 0.01 (**).

It was previously reported that TNFα regulates mitochondrial mass, which may be relevant in MPN patients^13^. The plasma TNFα concentration and platelet mitochondrial mass (expressed as number seen in 50 platelets by TEM) were higher in MPN patients than in healthy controls, and TNFα concentration was inversely correlated with mitochondrial mass in MPN platelets regardless of mutation (Fig. 2), which was also significant when this analysis was confined to *JAK2* V617F MPN (n = 15; r = − 0.75; *P* = 0.03).

**Figure 3:**
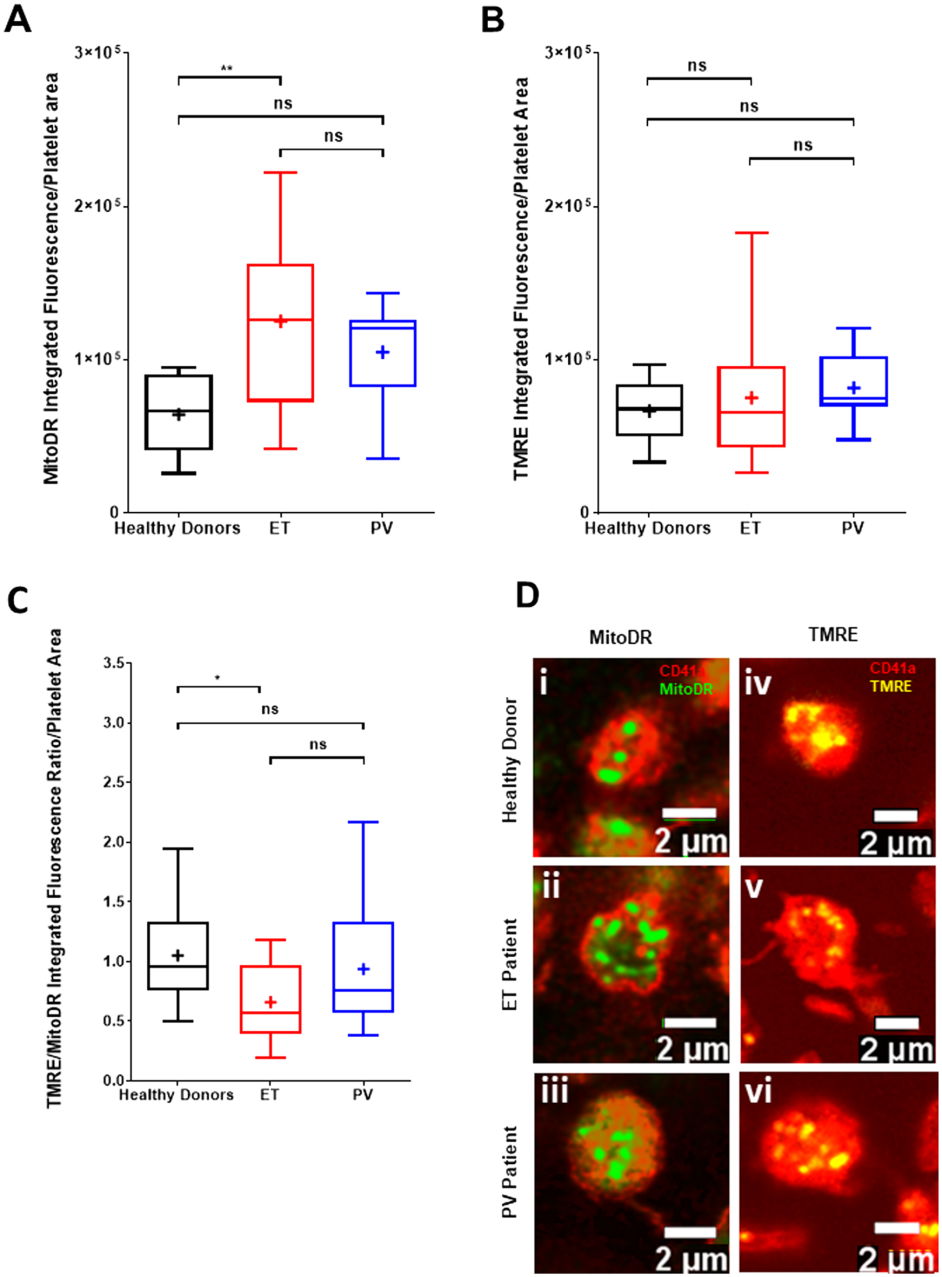
Confocal microscopy shows an increased proportion of dysfunctional mitochondria in resting platelets of ET patients. (**A**) Comparison of MitoDR staining of platelet mitochondria in healthy donors (n = 9), ET (n = 12) and PV (n = 8) patients as an indicator of mitochondrial mass within platelets. (**B**) Comparison of TMRE staining of platelet mitochondria as an indicator of functional mitochondria within platelets. (**C**) Comparison of TMRE/MitoDR ratios between healthy donors, ET and PV patients as an indicator of the number of functional mitochondria in proportion to overall mitochondrial mass within platelets. Bar-whisker plots, showing minimum to maximum values, median, interquartile range and mean (+), represent the spread of individual subject means per group. Data were analysed using a one-way ANOVA and Tukey’s multiple comparisons test. *P* < 0.05 (*), *P* < 0.01 (**) = significant, ns = not significant. (**D**) Representative confocal images showing the total number of mitochondria (i–iii) and the number of functional mitochondria (iv–vi) within platelets from healthy donors, ET and PV patients, respectively, as indicated by CD41a (red), MitoDR (green) and TMRE (yellow) staining.

MPN patients are known to have an increased immature platelet fraction^14^, which is associated with an increase in platelet volume. Since TEM provides only a planar section of the platelet, we used confocal microscopy to assess the density of mitochondria in the entire volume of the platelets corrected for platelet area (Fig. 3A). Separating MPN patients into ET versus PV, mitochondrial density (MitoDR staining) was significantly increased in ET patient platelets in the absence of agonist stimulation (median arbitrary fluorescence intensity/µm^2^ 126,107; n = 14) compared to healthy donor platelets (median 66,367; n = 10) while mitochondrial density in PV platelets was not significantly different from controls. Within the same individuals, the functional state of platelet mitochondria was assessed by TMRE, since level of TMRE fluorescence can be used to determine whether mitochondria in a cell have high or low ΔΨm. The integrated fluorescence intensity of TMRE staining per platelet did not differ between healthy donors (n = 18) and patients with ET (n = 12) or PV (n = 8; Fig. 3B). However, when corrected for the increased mitochondrial density, the proportion of functional mitochondria (expressed as a TMRE:MitoDR ratio) was significantly reduced in ET patients (mean = 0.66; median = 0.57; *P* = 0.016) compared to healthy donors (mean = 1.05; median = 0.96) with no significant difference in PV patients (mean = 0.94; median = 0.75; Fig. 3C). Representative images of MitoDR and TMRE staining of mitochondria in platelets of healthy donors, and ET and PV patients are shown in Fig. 3D.

**Figure 4:**
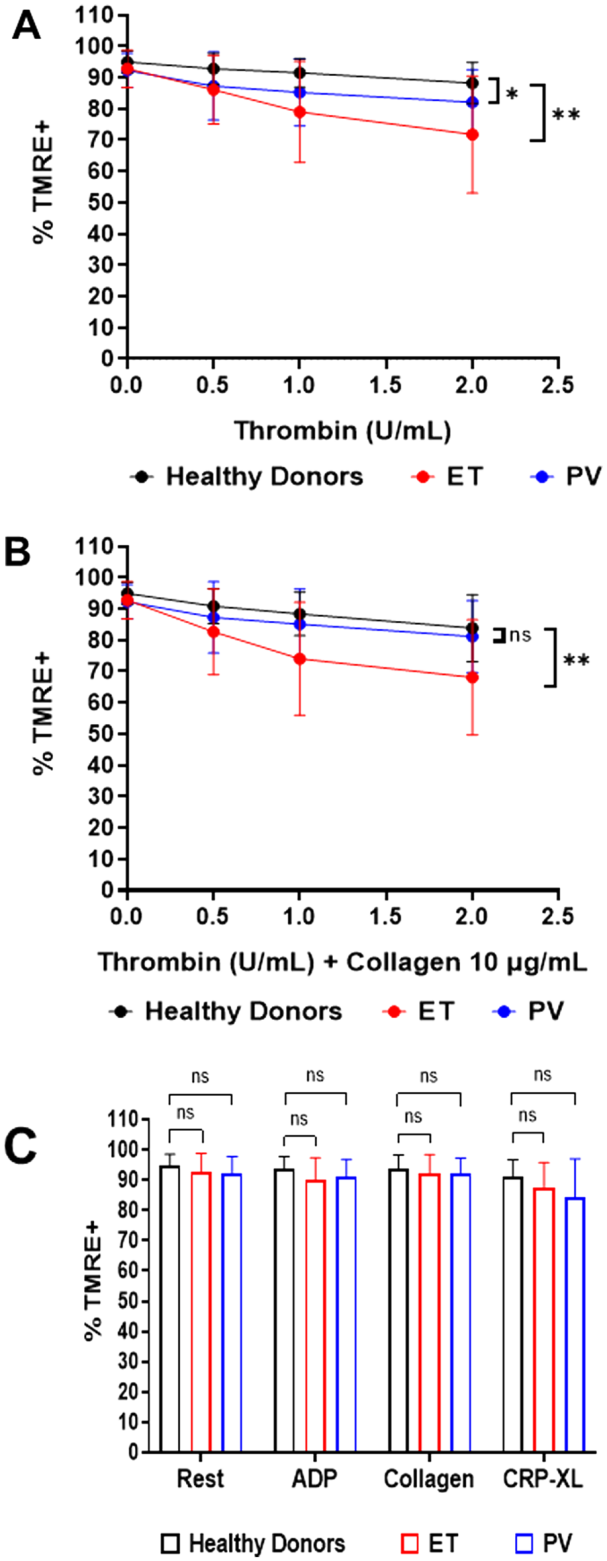
MPN platelet mitochondria are hypersensitive to depolarization following stimulation with thrombin. (**A**, **B**) Dose–response curves derived from stimulation with various concentrations of thrombin (**A**) or thrombin with collagen (10 µg/mL) (**B**) in healthy donors (n = 20) or ET (n = 35) and PV (n = 9) patients. Error bars for each point represent differences between individuals. Data analysis was by a mixed-effects 2-way ANOVA followed by Tukey’s multiple comparisons test. *P* < 0.05 (*), *P* < 0.01 (**) = significant, *ns* = not significant. (**C**) TMRE staining of mitochondria of healthy donors (n = 19–20), ET (n = 31–36) and PV (n = 9–11) patients under resting conditions, and following stimulation with ADP (5 mg/mL), collagen (10 µg/mL), or CRP-XL (1 µg/mL) alone. Column graphs showing error bars represent differences between individual subjects. Data were analysed using a one-way ANOVA or Kruskal–Wallis test. *P* < 0.05 (*) = significant, *ns* = not significant.

To determine whether MPN platelets were sensitized to loss of active mitochondria, platelets were assessed by flow cytometry for loss of TMRE staining in response to increasing doses of thrombin, with or without collagen 10 mg/mL. A significantly higher proportion of platelets from ET (n = 35) patients compared with healthy donors (n = 20) demonstrated TMRE loss at every dose of thrombin, with statistically greater differences with increasing thrombin doses (Fig. 4A , B). ET patients also demonstrated significant loss of ΔѰm compared with PV patients (n = 10) and both PV and ET platelets were significantly different from healthy donors (n = 20). In the presence of collagen, thrombin-induced platelet TMRE loss in ET patients was greater than in healthy donors, but not in PV patients (Fig. 4B). There was a non-significant trend to greater TMRE loss in JAK2 V617F ET (n = 21) compared with JAK2-negative ET (n = 14), (thrombin, *P* = 0.07; thrombin + collagen, *P* = 0.07: Supplementary Fig. S3 online). There was no statistically significant difference according to anti-thrombotic therapy (Supplementary Fig. S3C-F online). MPN platelets at rest or stimulated with weaker agonists, ADP, collagen, or GPVI agonist, cross-linked collagen-related peptide (CRP-XL), showed no difference in TMRE loss compared to healthy donor platelets (Fig. 4C). Similar to mitochondrial mass, TNFα did not correlate with loss of ΔѰm by TMRE staining.

**Figure 5:**
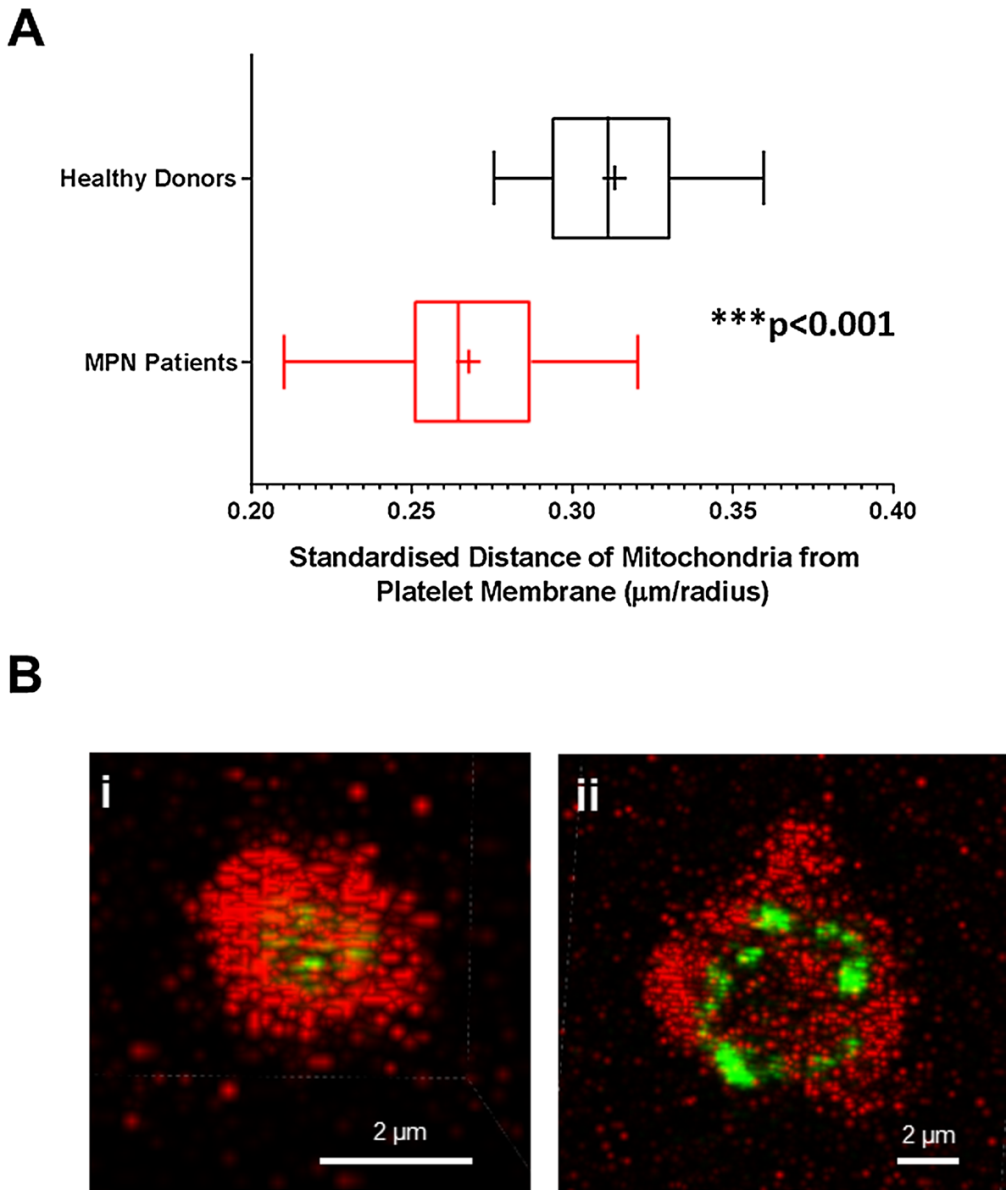
Platelet mitochondria from MPN patients are located nearer the surface membrane than in healthy donors. (**A**) Distance of mitochondria from the surface membrane (μm) was standardized by dividing by the mean platelet radius in platelets from healthy donors (n = 10) and MPN patients (n = 13). Box–Whisker plots showing the max, min, interquartile range, median and mean ( +) represent individual subject means. Mann–Whitney Test. *P* < 0.001 (***). (**B**) Representative confocal microscopy images showing differences in mitochondrial location in a healthy donor (i) and an MPN patient (ii)**.**

The spatial distribution of mitochondria in MPN platelets (n = 13) differed from that observed in healthy donors (n = 10). Distance from the platelet surface to each individual mitochondrion, expressed as a fraction of the radius in confocal images (Fig. 5) demonstrated that mitochondria in MPN platelets are located significantly nearer the platelet surface (median ratio 0.24, interquartile range 0.19–0.29) than in healthy donors (median ratio 0.28, interquartile range 0.24–0.34; *P* < 0.001). In TEM the absolute distance from the platelet surface (measured in nm) was not significantly different between MPN and healthy donors, possibly reflecting measurement in planar sections that was not corrected for platelet volume (Supplementary Fig. S4 online).

**Figure 6:**
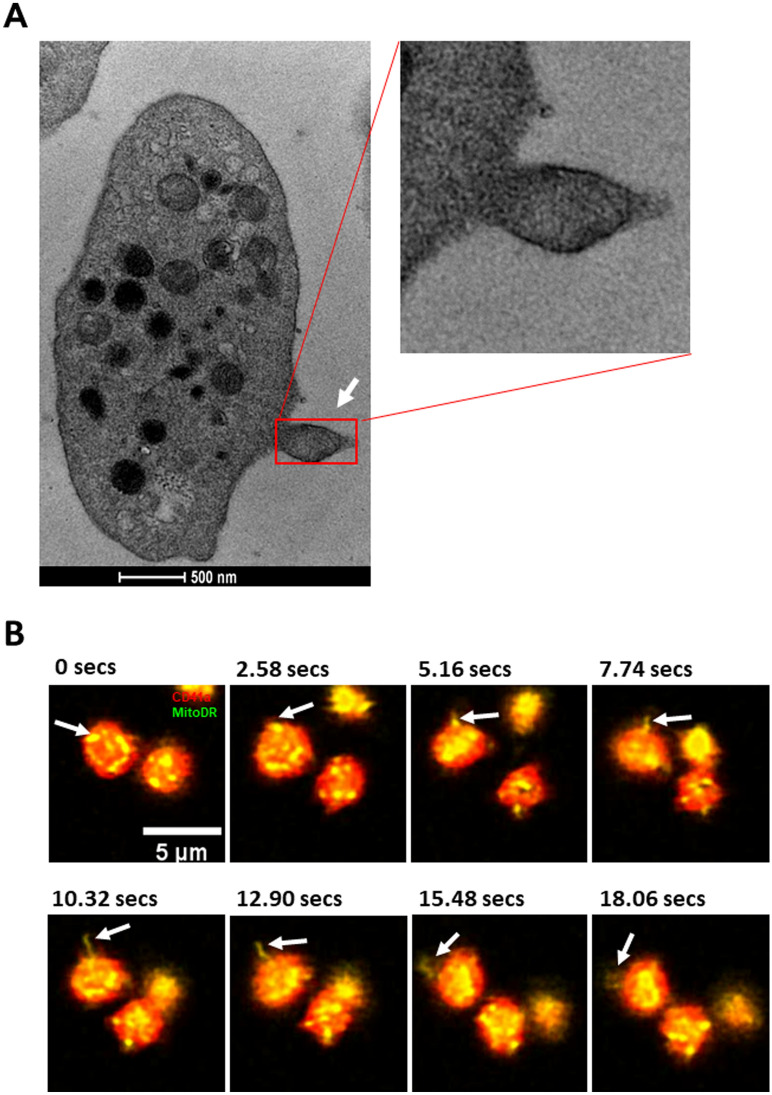
Mitochondria are extruded from platelets of MPN patients. (**A**) Representative TEM image of a mitochondrion (white arrow) being extruded from the platelet membrane, with enlargement. (**B**) Confocal microscope time lapse images showing a mitochondrion (white arrows) being extruded from a platelet of an MPN patient under resting conditions (CD41a = Red, MitoDR = Yellow).

TMRE loss could be due to extrusion of platelet mitochondria or depolarization of the mitochondria. TEM images of resting platelets were systematically examined for mitochondrial extrusion. In 26/1250 MPN platelets (2.1%) from 23 patients we were able to identify a mitochondrion in a pseudopodium (Fig. 6A) apparently in the process of being expelled from the platelet as a microparticle, whereas this appearance was seen in 4/339 platelets from 16 healthy donors (1.1%; *P* = 0.28). Mitochondrial extrusion was seen in live imaging by confocal microscopy only in resting MPN platelets (Fig. 6B), and not in healthy donor platelets.

**Figure 7:**
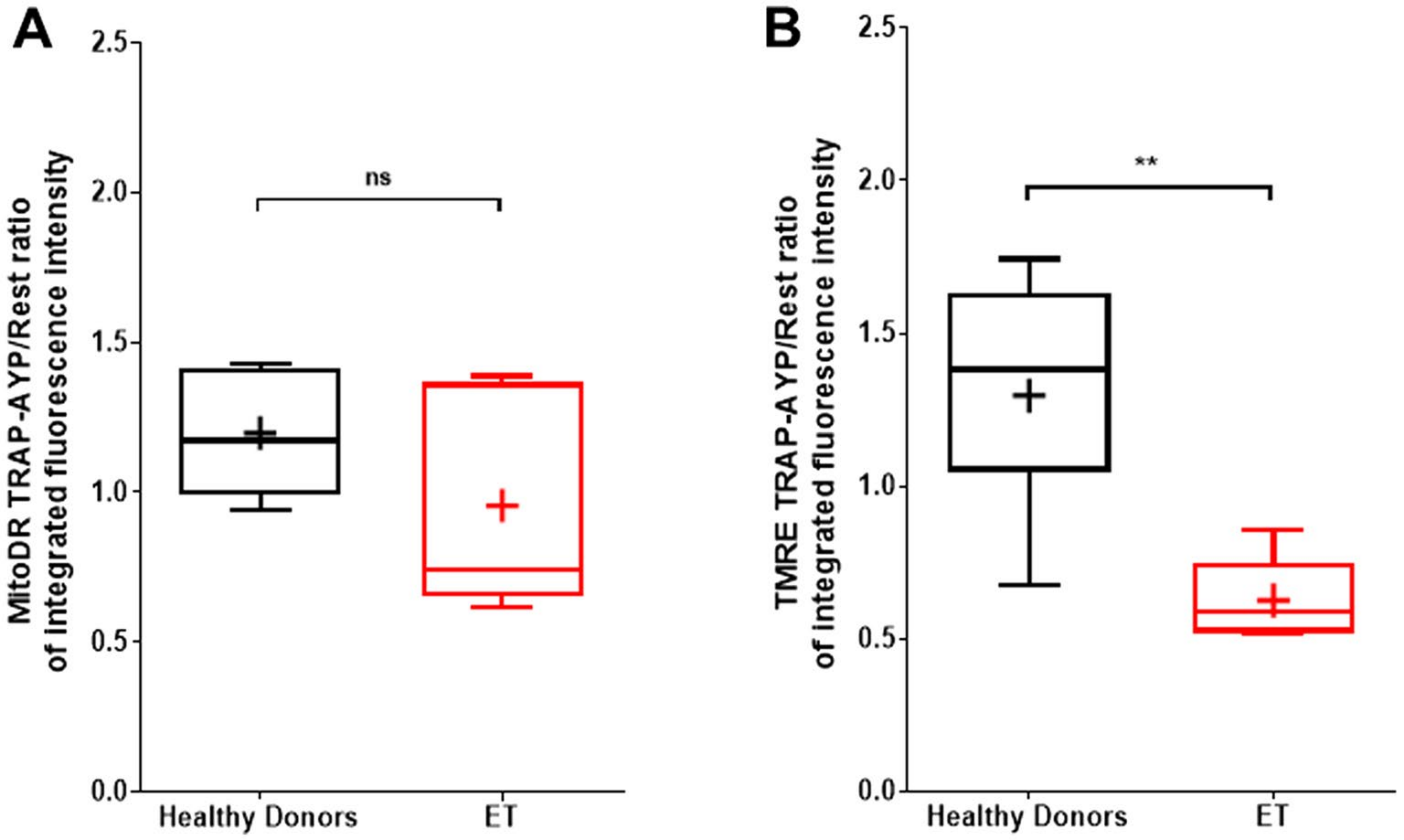
Loss of TMRE in ET platelets after agonist stimulation exceeds loss of MitoDR staining and occurs more rapidly than in healthy donors. (**A**) Confocal microscopy integrated fluorescence intensity of MitoDR staining expressed as a ratio of measurements before and after TRAP-AYP agonist stimulation (10 μM TRAP, 300 μM AYP, 1 mM calcium) shows no significant difference between healthy donors (n = 5–7) and ET patients (n = 5). (**B**) Integrated fluorescence intensity ratio of TMRE staining expressed as a ratio of measurements before and after TRAP-AYP agonist stimulation shows a significantly greater reduction in ET patients than in healthy donors. Box-Whisker plots showing the max, min, interquartile range, median and mean (+) represent individual subject means. Mann–Whitney Test. *P* < 0.01 (**).

Platelet mitochondrial density (MitoDR integrated fluorescence intensity) was determined before and after thrombin receptor stimulation using the combination of PAR1 and PAR4 agonists. The ratio of mitochondrial density before and after stimulation was not significantly different in ET patients (n = 5) compared with healthy donors (n = 7), but there was significantly greater TMRE loss in ET platelets following agonist stimulation (Fig. 7), indicating that depolarization of the mitochondrial membrane was the major cause of TMRE loss.

**Figure 8:**
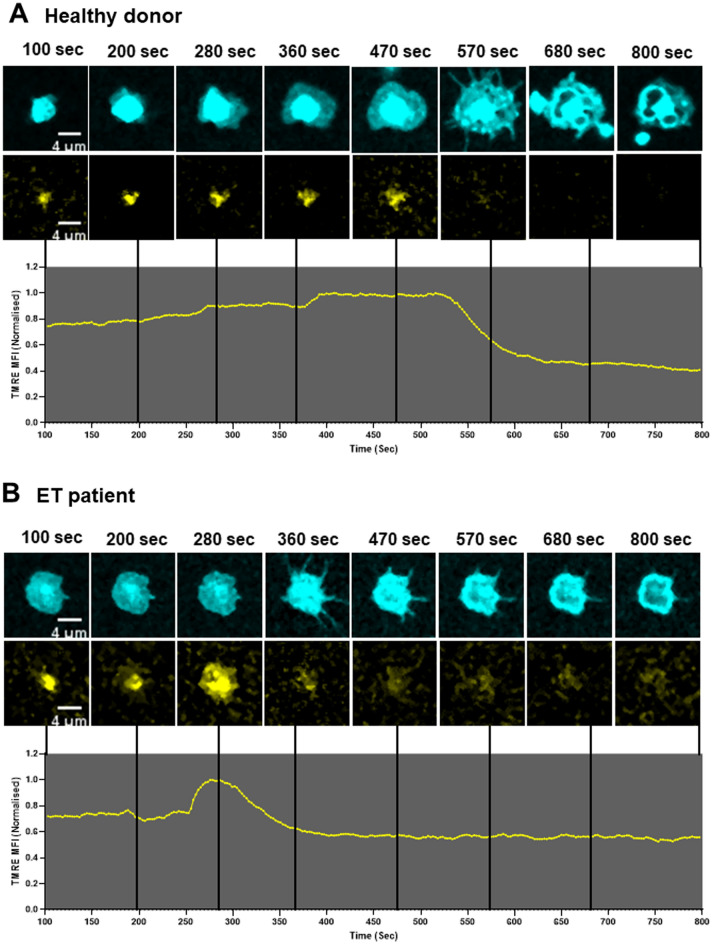

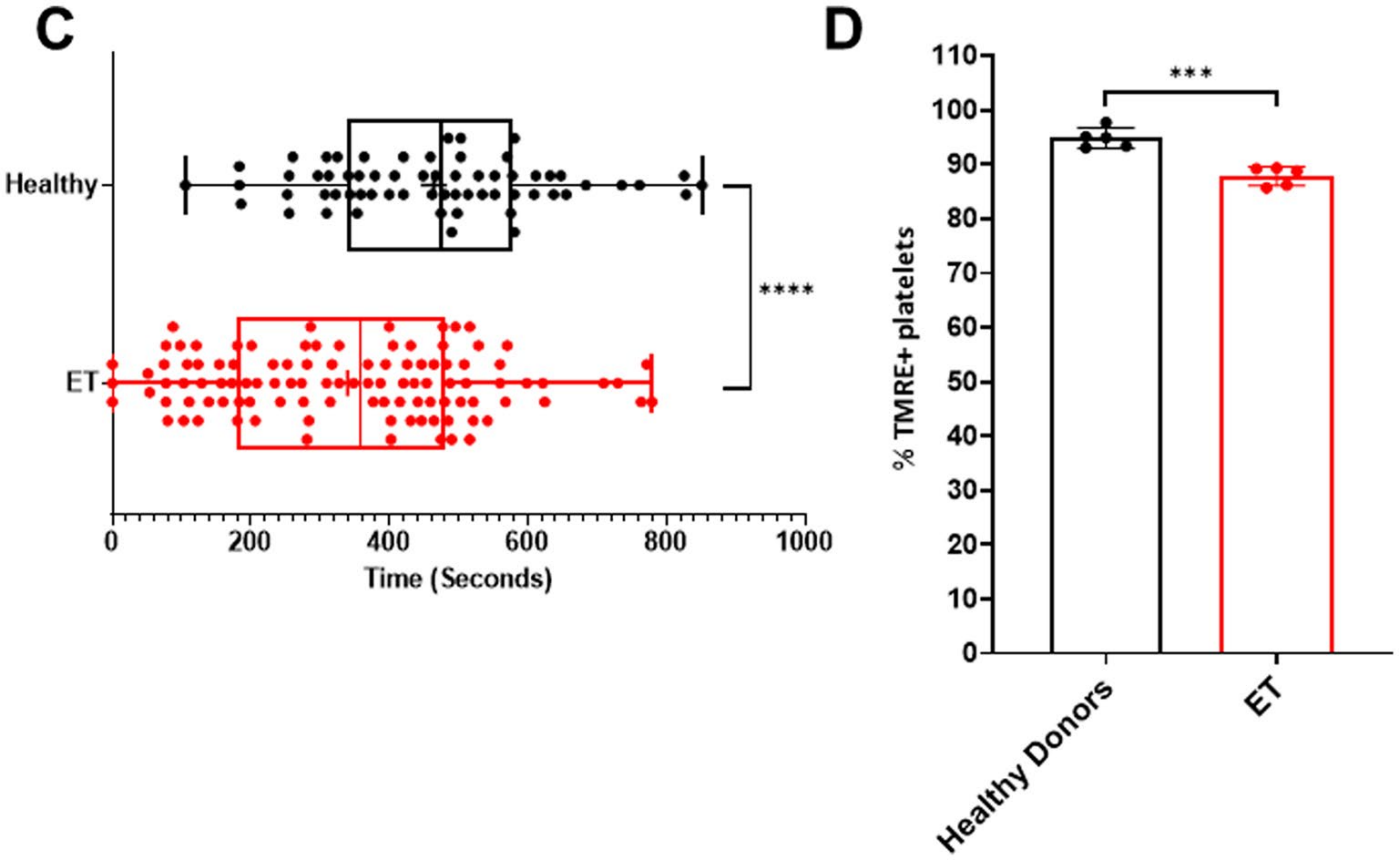
Mitochondrial depolarization precedes platelet ballooning and occurs earlier in ET patients than in healthy donors. (**A**) Representative graphical data and selected time lapse images of mitochondrial depolarization prior to platelet balloon formation as indicated by TMRE loss in a healthy donor and (**B**) in an ET patient. Time lapse images begin at 100 s after addition of agonist (thrombin 2 U/mL and calcium 1 mM). TMRE mean fluorescence intensity values were normalized to the highest intensity. Line graphs representing TMRE loss were subjected to exponential smoothing with a dampening factor set at 0.8 to remove background noise. (**C**) Confocal microscopy analysis of time lapse images comparing healthy donors and ET patients showing time to mitochondrial depolarization (TMRE loss) following stimulation with thrombin (2 U/ml) and calcium (1 mM). Box–Whisker plots showing time values from individual platelets: max, min, interquartile range, median and mean ( +) from 5 healthy donors (n = 63 platelets) and 5 ET patients (n = 106 platelets); unpaired t-test, *P* < 0.0001 (****). (**D**) Endpoint analysis of confocal time lapse images comparing the percentage of platelets with TMRE retained in healthy donors (n = 5) and ET patients (n = 5). Error bars on column graphs represent standard error of the mean and dots represent individual subject values (Healthy donors, range: 134–289 platelets/individual; ET patients, range: 102–232 platelets/individual); unpaired t-test, *P* < 0.001 (***).

Visualization of platelet mitochondria by live microscopy following thrombin stimulation demonstrated loss of the TMRE signal consistent with mitochondrial depolarization rather than mitochondrial extrusion as the dominant feature. High resolution continuous imaging showed TMRE loss occurring sequentially in adjacent mitochondria within individual platelets (Supplementary Fig. S5 and video online). Platelet ballooning followed loss of TMRE staining and was not observed in platelets that retained TMRE (Fig. 8A, B; Supplementary Fig. S5 and video online).

Loss of TMRE occurred earlier in ET (n = 106 platelets, 5 patients) than in healthy donor platelets (n = 63 platelets, 5 donors) after a mean of 131.8 ± 14.1 s versus 180.2 ± 16.2 s (*P* < 0.0001; Fig. 8C). The proportion of platelets with TMRE loss 15 min after thrombin stimulation was significantly greater in ET (mean 12.1% ± 1.8) than in healthy donor platelets (mean 5.1% ± 1.9; *P* < 0.001; Fig. 8D) with no additional TMRE loss seen with imaging time extended to 1 h.

## Discussion

Although conventional treatment targets in ET and PV relate to normalized blood cell counts, there is only limited correlation between the platelet count and clinical thrombosis risk. *JAK2* V617F clonal hematopoiesis without clinical evidence of a myeloid neoplasm is in itself associated with an increased risk of thrombosis, even when the mutation is present at a low VAF^15, 16^. These observations suggest that cell-intrinsic alterations, as well as elevated blood counts, play a role in thrombosis risk.

We have shown that mitochondrial mass is increased in MPN platelets. This is consistent with historical observational studies performed prior to the discovery of driver mutations^17, 18^, and a recent report of increased mitochondria in MPN, involving 9 patients with *JAK2* V617F^13^. The authors showed that mitochondrial mass in ageing mice was positively correlated with TNF-α concentration, and reduced by TNF-α blockade^13^. TNF-α levels are commonly increased in MPN, with evidence that the elevation in TNF-α is more pronounced in patients with *JAK2* V617F^19^. Although we confirmed the observation that both TNF-α concentration and mitochondrial mass are increased in *JAK2* ET, we also observed increased mitochondrial mass in *CALR* and triple-negative MPN patients in whom TNF-α levels were lower. Contrary to expectation, we found an inverse correlation between *JAK2* V617F VAF and mitochondrial mass in MPN. The highest TNF-α concentrations were seen in patients with myelofibrosis, so it is possible that the mitochondrial mass is influenced by the histological MPN subtype. There were numerically more mitochondria in platelets from ET patients than from other MPN subtypes, but our study was not adequately powered to assess the effect of multiple clinical parameters. A recent meta-analysis demonstrated substantial heterogeneity in studies of sex-related differences in mitochondrial number and function^20^, highlighting the importance of adequately powered studies. Nevertheless, our data indicate that factors other than *JAK2* mutation and TNF-α levels contribute to variation in mitochondrial mass in MPN.

We found substantial heterogeneity in mitochondrial mass between individual platelets from individual MPN patients. Since the platelets with markedly increased mitochondrial content represented only a small fraction, it is possible that this is a stochastic phenomenon in the process of platelet formation. However, it is also possible that the heterogeneity of mitochondrial content could relate to the clonality of individual megakaryocytes from which the platelets were derived. We found no correlation between the VAF of *JAK2* V617F (measured in leukocyte DNA) and mitochondrial mass in platelets. Platelet clonality could not be assessed in this study, but previous studies have shown that not all platelets in an MPN patient are derived from the MPN clone^21^, which is especially relevant in ET in which the VAF of *JAK2* V617F is typically lower than in PV and MF^22^.

Changes in platelet function in MPN are complex with an apparent paradox involving variable reduction in platelet reactivity (platelet exhaustion) co-existing with an increase in hypercoagulability and platelet-dependent thrombin generation^23, 24^. Although platelets from ET patients had more mitochondria, resting ET platelets had lower ΔѰm than healthy controls, which suggests reduced oxidative phosphorylation activity at baseline. Given that mitochondrial depolarization is a hallmark of necrosis^25^, our observation may help explain the mechanism of platelet exhaustion in MPN^24^, acknowledging that we did not specifically measure exhaustion in this study.

In line with prior reports of platelet hypersensitivity to thrombin stimulation in MPN^26^, ET patients also demonstrated increased numbers of platelets with loss of TMRE after stimulation with thrombin or thrombin plus collagen, compared with healthy donors. We speculate that baseline partial mitochondrial membrane depolarization sensitized ET platelets to increased and more rapid complete depolarization in response to thrombin stimulation. Of note, platelet mitochondrial depolarization in healthy individuals is a feature of co-stimulation of the protease-activated receptor and immunoreceptor tyrosine activation motif (ITAM) pathways. Previous studies demonstrating Src tyrosine kinase activity associated with platelet hypersensitivity in both ET and PV suggest that the ITAM pathway may already be pre-activated in MPN^3^, accounting for our observation that the sensitization to mitochondrial depolarization was limited to thrombin stimulation in this MPN cohort with no synergistic increase with collagen.

Mitochondrial depolarization occurred more rapidly after thrombin stimulation in ET, and in a stochastic fashion—the majority of platelets had no change in TMRE staining, but in platelets with TMRE loss, an ‘all-or-nothing’ phenomenon was seen in live imaging with all mitochondria within those platelets undergoing depolarization within seconds after a variable latency period. Depolarization is associated with permeabilization of the mitochondrial membrane and causes a spike in cytoplasmic calcium which can, in turn, lead to depolarization of adjacent mitochondria^10^. When the density of mitochondria within the platelet is increased, this would be predicted to enable more efficient propagation of calcium flux to adjacent mitochondria^27^. Consistent with this hypothesis, thrombin hypersensitivity to depolarization was most marked in the cohort of patients with ET (versus PV) in whom mitochondrial mass showed the greatest increase. We speculate that the platelets most susceptible to mitochondrial depolarization are the subset with the highest mitochondrial number. Formal testing of this hypothesis would require studies of calcium flux in single platelets with variable mitochondrial content.

A further consequence of calcium flux and mitochondrial membrane depolarization is the exposure of phosphatidylserine on the surface of platelets, providing a substrate for activation of coagulation^10, 25^. TMRE loss is one of the characteristics of procoagulant platelets^28^, and we demonstrated that TMRE loss was followed by the characteristic membrane ballooning of procoagulant platelets^29^. This observation suggests that excess procoagulant platelets may be an additional prothrombotic mechanism in MPN. The increased loss of viable platelets linked with increase in procoagulant platelet membrane surface is a potential link with the phenomena of hypercoagulability, despite findings of reduced classical platelet activation in MPN.

Several studies have reported increased procoagulant microparticles in MPN^30–32^, but none has shown a specific role of cell-free mitochondria. In models of transfusion-related acute lung injury and brain injury it has been shown that cell-free mitochondria are released and have a pro-inflammatory, procoagulant effect. Some procoagulant microparticles contain mitochondria and some are bare mitochondria^11^. We showed that mitochondria in MPN platelets tend to be peripherally located, which may facilitate their shedding as microparticles. We directly observed mitochondria in platelet pseudopodia, more often in MPN samples than in healthy controls, although our study was not powered to detect a statistically significant difference in the frequency of this rare event.

This pragmatic study has some significant limitations. The sample size in individual experiments exploring mitochondrial number, location, and function did not permit us to explore whether any of these differences were associated with clinical thrombotic events, therapy, or MPN subtype. The full range of mitochondrial testing could not be performed on a single sample for each patient, so sequential cohorts were recruited for each experimental set, along with contemporaneous control samples.

Platelet mitochondrial dysfunction is increasingly recognized as a contributor to pathological thrombosis^33^. We have shown that mitochondrial mass is increased in MPN platelets, that mitochondrial membrane potential is altered prior to stimulation in circulating platelets, and that MPN platelets are hypersensitive to depolarization of the mitochondrial membrane and formation of balloon platelets. Furthermore, mitochondria may have a greater propensity to be expelled from MPN platelets as microparticles. Further study is needed to determine whether these phenomena relate to disease subtype or clinical thrombosis risk in MPN.

## Methods

### Patients

MPN patients and healthy donors were recruited in three hospitals and gave written, informed consent. The study protocol was approved by the relevant Human Research Ethics Committees (Sydney Local Health District HREC/2019/ETH08002 and Central Adelaide Local Health Network HREC/2012/RAH189). All research was performed in accordance with relevant guidelines/regulations and the Declaration of Helsinki. Healthy donors were aged over 20 years with no history of coagulation disorders and no personal history of MPN. Patients had a diagnosis of a classic MPN, irrespective of treatment. Patients on thrombin inhibitors were excluded from experiments in which thrombin was used as an agonist.

### Blood collection

Blood was collected with the use of a tourniquet into evacuated tubes with citrate or EDTA anticoagulants. Platelet-rich plasma (PRP) for transmission electron microscopy (TEM) was prepared by centrifugation of citrate-anticoagulated whole blood at 400–1600 g for 5 min or 200 g for 10 min (depending on the experimental protocol) with slow acceleration and no braking. Plasma TNF-α concentration was measured by enzyme-linked immunosorbent assay using a Quantikine™ HS ELISA human TNFα immunoassay (R&D Systems, Minneapolis, USA) according to the manufacturer’s instructions.

### Transmission electron microscopy

PRP was fixed in 1% glutaraldehyde and then centrifuged at 2600 g for 10 min to form a pellet. The platelet pellet was fixed in sodium cacodylate and osmium tetroxide. Ultrathin sections were viewed using a Technai G2 Spirit EM (FEI, Hillsboro, Oregon, USA).

### Confocal microscopy

Mitochondrial mass was assessed by confocal microscopy using MitoTracker red CMXRos (Invitrogen, Eugene, OR, USA), a stain that accumulates in active mitochondria with intact membrane potential and CellMask Deep Red Plasma Membrane (Life Technologies, Carlsbad, CA, USA) to delineate the outer platelet membrane. PRP was incubated with the stains at room temperature in the dark for 5 min. Washing steps were omitted to avoid platelet activation. Platelets were immobilized in 1% agarose gel and images were acquired using a Nikon Eclipse Ti A1R inverted microscope. Z-series optical sections were collected with a step-size of 0.175 microns and 3-dimensional deconvolution was performed on all z-stacks.

Mitochondrial membrane potential (ΔѰm) was assessed by confocal microscopy using tetramethylrhodamine ethyl ester perchlorate (TMRE; Sigma-Aldrich, Sydney, Australia). PRP was diluted in Hank’s buffered saline solution (HBSS) to a concentration of 1 × 10^4^ platelets/µL, labelled with platelet marker CD41a-BV421 (BD Biosciences, San Diego, CA, USA) and either MitoTracker Deep Red (MitoDR) (Thermo-Fisher Scientific, Melbourne, Australia) or TMRE at 37 C for 15 min and transferred to an 8-well chamber slide (Ibidi, Gräfelfing, Germany). For experiments involving non-adherent platelets, slides were pre-coated with 5 mg/mL of bovine serum albumin. Platelets were imaged at rest or after agonist stimulation in the presence of fibrin polymerization inhibitor GPRP, 2 mM (Sigma‐Aldrich) plus calcium, 1 mM at set time points and by continuous video microscopy. Agonists were either thrombin (2 U/mL), or combination PAR1 agonist (TRAP, 10 µM) with PAR4 agonist AYPGKF (AYP, 300 µM). For experiments involving adherent platelets and observing mitochondrial depolarization and platelet ballooning, 8-well chamber slides were pre-coated with 50 µg/mL of collagen. Platelets were stained as previously described followed by agonist stimulation with thrombin (2 U/mL) and 1 mM calcium. Live-image capture, in a 1048 × 1048 pixel field of view, commenced approximately 30 s after agonist addition and was run for 1 h at a frame rate of 2.578 s with endpoint analysis at 15 min. Imaging of stained platelets was performed using a Leica TCS SP8 confocal microscope with either an HC PL APO 63x/1.40 OIL CS2 or HC PL APO 63x/1,20 W CORR CS2 objective lens. Post-acquisition endpoint image and video analysis to obtain mean intensity, integrated density of stained mitochondria, platelet area values and mitochondrial depolarization curves was performed using Fiji/ImageJ software. All platelets in a standardized field were masked and numbered in Image J for tracking. Mitochondria were individually masked and mean fluorescence intensity of TMRE was measured at each time point until 15 min was reached. Time at which TMRE fluorescence returned to background was taken as time to TMRE loss. Morphological change to balloon phenotype was determined visually in tracked platelets within the video analysis.

### Flow cytometry

Platelet ΔѰm was measured by flow cytometry in citrated whole blood using TMRE. Briefly, 15 μL of whole blood was diluted with Hanks’ balanced salt solution (HBSS, pH adjusted to 7.35), final concentrations of 2.5 mM GPRP and 2.5 mM calcium—with and without agonists. Reactions were stopped after 10 min of incubation at room temperature by a 20‐fold dilution with HBSS, aliquots labelled for 15 min with fluorescent tagged antibodies. The antibody mix consisted of the TMRE dye, CD41a (HIP8, BD Biosciences) and CD45 (HI30, StemCell Technologies, Vancouver, Canada). Samples were resuspended in 1 mL of HBSS buffer with 0.35% (v/v) human serum albumin, immediately analysed on a BD LSRFortessa with acquisition of 10,000 platelet events. Gating was performed as previously described for procoagulant platelet enumeration^34, 35^. Analysis was performed using FlowJo™ software (BD Biosciences, Ashland, Oregon).

### Statistical analysis

Confocal microscopy and TEM raw data were averaged for each healthy donor control and MPN patient, and graphs plotted as a mean of means. This was done for the raw values of integrated density, integrated density ratio, mitochondrial distance from membrane/radius and number of mitochondria per platelet. Control and patient averages were tested for normality using the Shapiro–Wilk test and homogeneity of variance was determined using Bartlett’s test. Statistical differences between groups were determined using either a parametric unpaired, two-tailed t-test for comparison of two groups or a one-way ANOVA for comparison of three or more groups. ANOVA *post-hoc* analysis was performed using Tukey’s multiple comparisons test. Flow cytometry data were analysed by comparing %TMRE expression between the categorical variables of healthy donors, ET patients and PV patients, and the continuous variable of thrombin concentration using a two-way ANOVA mixed-effects model. ANOVA *post-hoc* analysis was performed using Tukey’s multiple comparisons test. Correlation was performed using Pearson correlation. Statistical significance was set at *P* < 0.05.

## Supplementary Information

Supplementary Information.

## Data Availability

Requests for access to primary data should be addressed to the corresponding author.
