# Supplementary material for: Platelets from patients with myeloproliferative neoplasms have increased numbers of mitochondria that are hypersensitive to depolarization by thrombin
Source: Sci Rep. 2023 Jun 6;13:9172. doi: 10.1038/s41598-023-36266-2 (PMC10244326; doi:10.1038/s41598-023-36266-2)
Supplement: Supplementary file 1 — Supplementary Information. [file 41598_2023_36266_MOESM1_ESM.docx]

**Supplementary Materials**

**Supplementary Table 1. Characteristics of MPN patients**

| Median age | 65 years (range, 30-96) |
| --- | --- |
| Sex (female:male) | 46:43 |
| Diagnosis (ET:PV:MF^a^) | 57:22:10 |
| Median time since diagnosis | 30 months (range, 0-325) |
| Prior thrombosis | 27 (31%) |
| Arterial^b^ | 18 |
| Venous | 7 |
| Both | 2 |
| Driver mutation type^c^ |  |
| *JAK2* V617F | 60 |
| *CALR* exon 9 | 15 |
| *MPL* exon 10 | 1 |
| Triple-negative | 9 |
| Median platelet count | 452 x 10^9^/L (range, 80-1660) |
| Cytoreductive treatment | 56 (64%) |
| Hydroxyurea | 38 |
| Anagrelide^d^ | 8 |
| Other^e^ | 10 |
| Anti-thrombotic treatment | 81 (93%) |
| Aspirin^f^ | 65 |
| Anticoagulant^g^ | 12 |
| Clopidogrel | 4 |

1. Including PMF (n=8) and post-ET and post-PV MF (n=1 each).
2. Including cardiac (n=10) or cerebrovascular (n=9) ischemia, and peripheral vascular disease (n=5). Some patients had involvement of multiple arterial territories.
3. Mutation status unknown (n=2); *JAK2* V617F negative without further testing (n=2)
4. Anagrelide treatment was for ET (n=7) and PV (n=1).
5. Other cytoreductive agents were ruxolitinib (MF, n=5), interferon-α (ET, n=3; PV, n=1), and busulfan (ET, n=1).
6. Including 4 patients on a second anti-platelet drug (ticagrelor, n=2; clopidogrel, n=1; dipyridamole, n=1).
7. Including 4 patients on an anti-platelet drug as well as an anticoagulant. The anticoagulants used were warfarin (ET, n=3; MF, n=2; PV, n=2), dabigatran (PV, n=2), apixaban (ET, n=1; PV, n=1), and rivaroxaban (PV, n=1).

**Supplementary Figure 1: Confocal microscopy shows increased mitochondria in platelets of MPN patients.** The number of mitochondria in 50 platelets was counted using MitoDR staining for each individual subject. A comparison between healthy donors (n=10) and MPN patients (n=13) of the number of mitochondria per platelet was done using categorized data ranging from 0-20 mitochondria/platelet. Error bars at each point represent differences between individuals.


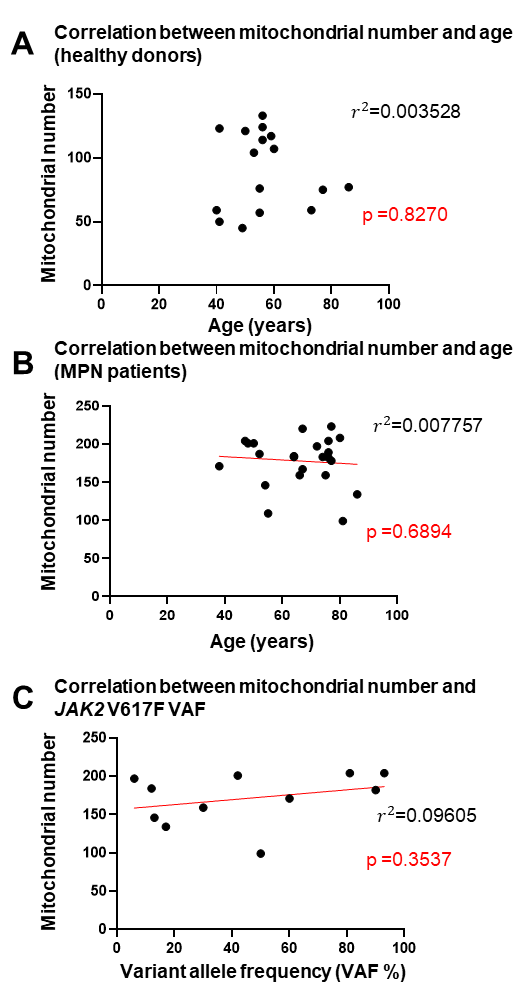


**
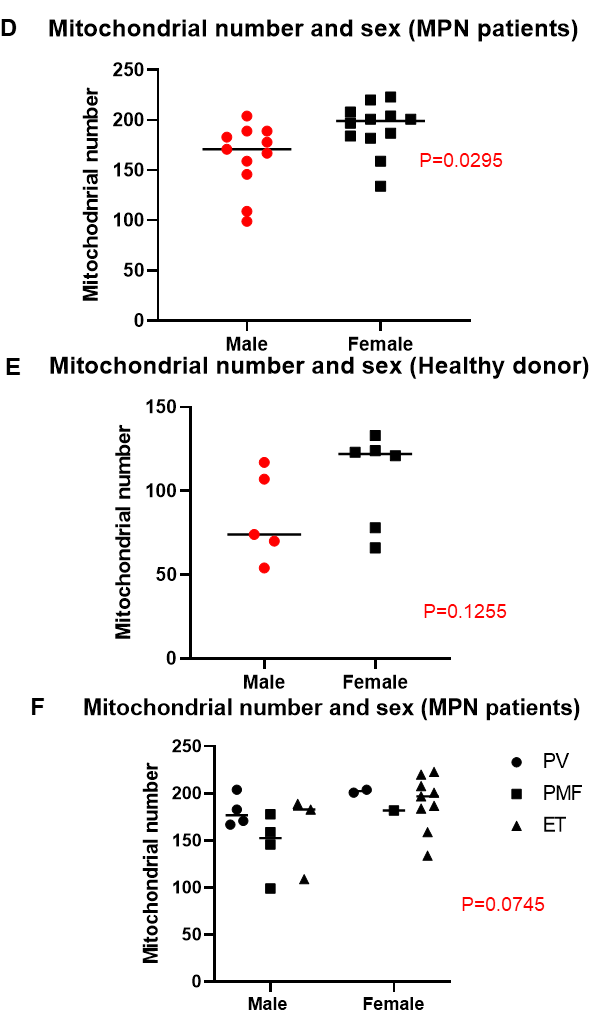
**

**Supplementary Figure 2. TEM assessment of mitochondrial number according to age, JAK2 mutation VAF, sex, and MPN subtype.** The total number of mitochondria in 50 platelets was plotted against age in **(A)** healthy donors (n=16) and **(B)** MPN patients (n=23). **(C)** The total number of mitochondria in 50 platelets was plotted against leukocyte VAF of *JAK2* V617F in MPN patients with that mutation (n=11). Pearson correlation. The total number of mitochondria in 50 platelets was plotted against according to sex in **(D)** healthy donors (n=10) and **(E)** MPN patients (n=23). Unpaired t-test. Panel **(F)** shows the number of mitochondria according to sex and MPN subtype. Two-way ANOVA test.

**Platelet TMRE Loss following Thrombin Stimulation in MPN Patients**

**A**

**B**

**
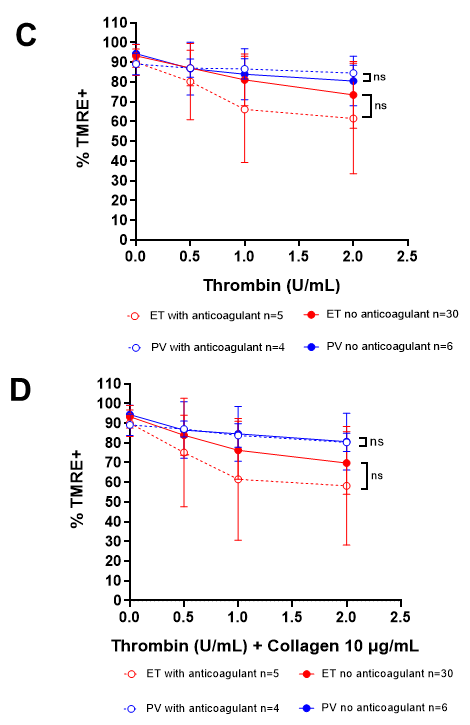
**

**E**

**F**

**Supplementary Figure 3. Platelet TMRE loss following stimulation with thrombin was greater but not significantly different in JAK2+ MPN patients compared to JAK2- MPN patients.** Dose-response curves derived from stimulation with various concentrations of thrombin **(A)** or thrombin with collagen (10 µg/mL) **(B)** in JAK2+ (n=21) and JAK2- (n=14) MPN patients. Data analysis was by a mixed-effects 2-way ANOVA followed by Tukey’s multiple comparisons test. The effect of patient treatment with or without an anticoagulant was examined for **(C)** dose response to thrombin without collagen, and **(D)** dose response to thrombin with collagen (10 mg/mL). Data analysis was by mixed-effects 2-way ANOVA followed by Šídák’s multiple comparisons test. The effect of patient treatment with (n=6) or without (n=29) P2Y12 inhibitors was examined for **(E)** dose response to thrombin without collagen, and **(F)** dose response to thrombin with collagen (10 mg/mL). Data analysis was again by mixed-effects 2-way ANOVA followed by Šídák’s multiple comparisons test. Error bars for each point represent differences between individuals. *ns* = not significant.

**Supplementary Figure 4.** **Mitochondrial location within healthy donors and MPN patients using TEM.** Absolute distance (nm) of mitochondria form platelet outer membrane assessed by TEM does not differ between healthy donors (n=16) and MPN patients (n=23). Bar-Whisker plots showing the max, min, interquartile range, mean. median and mean (+) represent individual subject means. Mann-Whitney Test, P = 0.20.


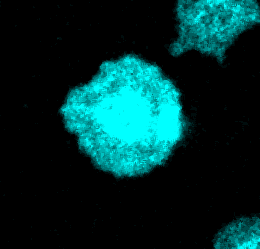

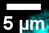

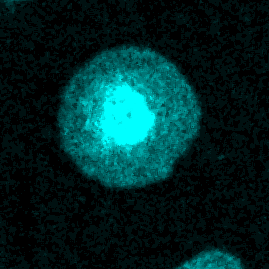

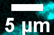

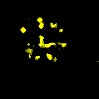

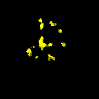

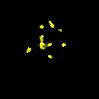

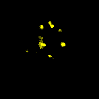

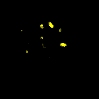

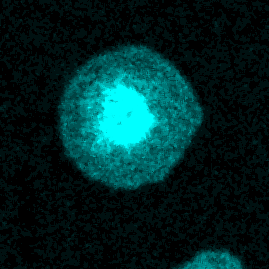

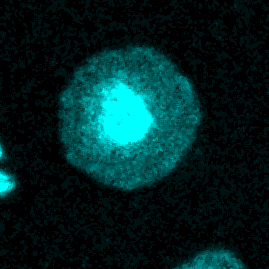

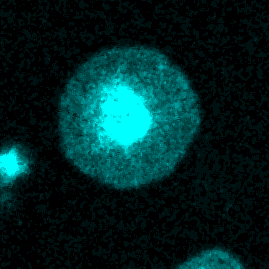

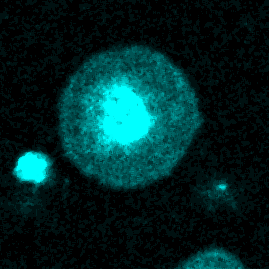

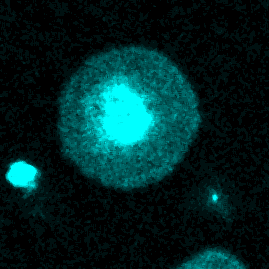

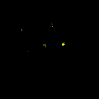

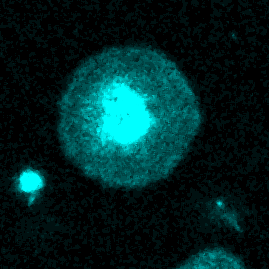

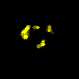

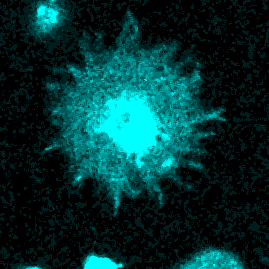

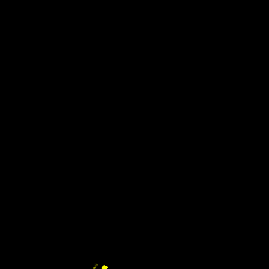

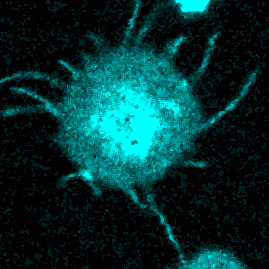


**43 sec**

**59 sec**

**92 sec**

**100 sec**

**105 sec**

**108 sec**

**113 sec**

**154 sec**


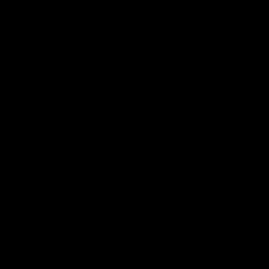


**216 sec**


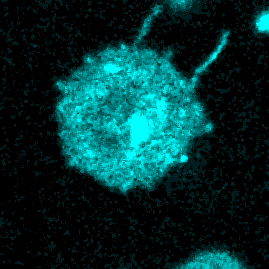

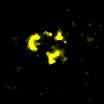

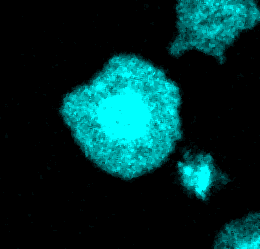

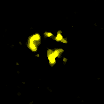

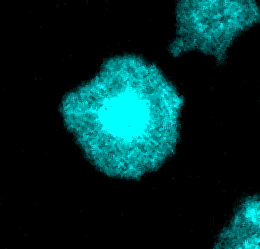

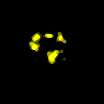

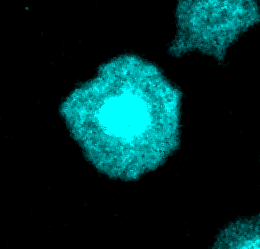

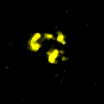

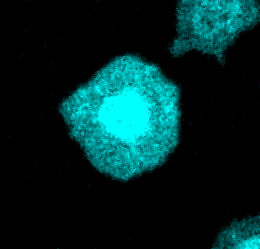

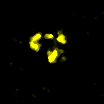

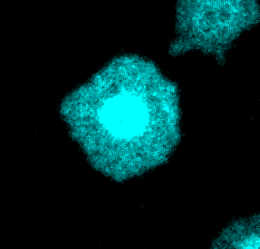

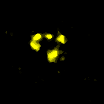

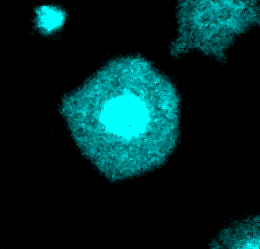

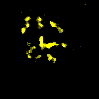

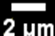

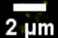

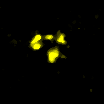

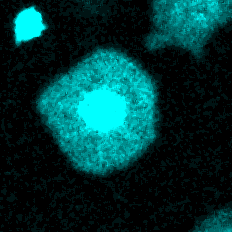

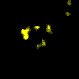

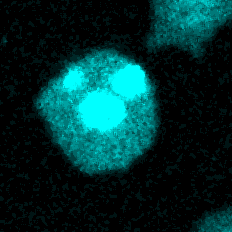


**A**

**B**

**CD41a**

**TMRE**

**CD41a**


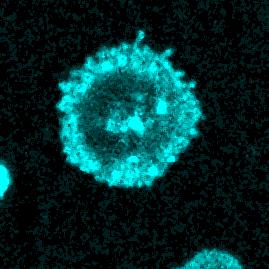

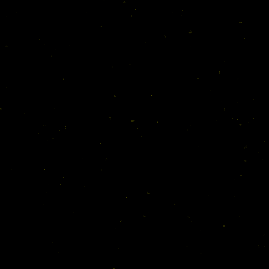


**747 sec**


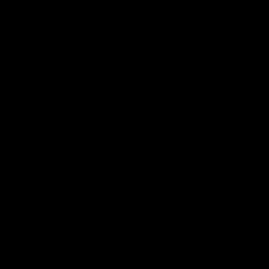


**391 sec**


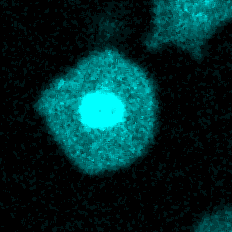

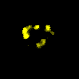

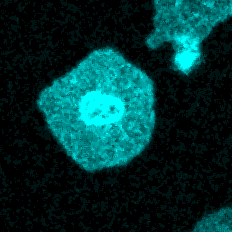

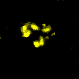


**TMRE**

**Timelapse Images of Mitochondrial Depolarization in an MPN Patient Following Thrombin Stimulation**

**Spread platelet (lamellipodium)**

**Pre-ballooning**

**platelet with**

**filipodia**

**Early balloon**

**formation**

**Fully ballooned**

**platelet**

**Supplementary Figure 5.** **High-resolution representative timelapse images of mitochondrial depolarization in an MPN patient following thrombin stimulation.** **(A)** Timelapse images from live microscopy of a single representative platelet from an MPN patient showing sequential TMRE loss in adjacent mitochondria prior to balloon formation following the addition of agonist (thrombin 2U/mL + calcium 1mM). **(B)** Stable platelet from same field of view as **(A)** over the same time course showing no TMRE loss or ballooning. Initial timelapse imaging begins at 20 seconds after addition of agonists thrombin and calcium.

**Video A**

**Video B**


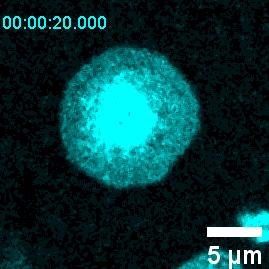

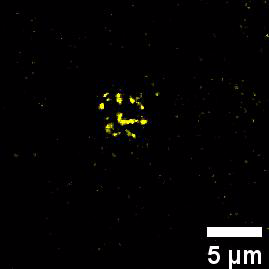

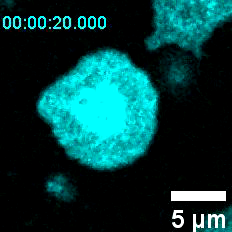

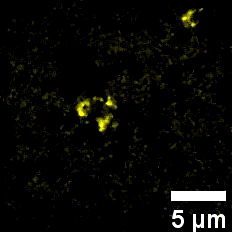


**Supplementary video: Live video microscopy of platelets A and B from Supplementary figure 5.**

**TMRE**

**CD41a**

**TMRE**

**CD41a**

**Supplementary Video. Visualization by live microscopy of mitochondrial depolarization within a platelet from an MPN patient following thrombin stimulation.** High-resolution videos of a platelet from an MPN patient at various stages of mitochondrial depolarization showing sequential TMRE loss in adjacent mitochondria prior to balloon formation following the addition of agonist (thrombin 2U/mL + calcium 1mM). Initial timelapse imaging begins at 20 seconds after addition of agonists thrombin and calcium.
